# Deep learning for fluorescence confocal microscopy image interpretation in radical prostatectomy

**DOI:** 10.1111/bju.70273

**Published:** 2026-04-19

**Authors:** Lixiang Fang, Nikhil Mayor, Alexander Light, Anna Silvanto, Aiman Haider, Chase Ng, Archana Gopalakrishnan, Ranil Johann Boaz, Mariana Bertoncelli Tanaka, Bijan Khoubehi, Giles Hellawell, Ricardo Almeida‐Magana, Larissa Mendes, Eoin Dinneen, Greg Shaw, Ben Challacombe, Paul Cathcart, Martin J. Connor, Taimur T. Shah, Hashim U. Ahmed, Francesca Fiorentino, Stamatia Giannarou, Mathias Winkler

**Affiliations:** ^1^ Division of Surgery, Department of Surgery and Cancer, Faculty of Medicine Imperial College London London UK; ^2^ Imperial Prostate, Division of Surgery, Department of Surgery and Cancer, Faculty of Medicine Imperial College London London UK; ^3^ Department of Urology Imperial College Healthcare National Health Service (NHS) Trust London UK; ^4^ Department of Pathology University College London Hospitals NHS Foundation Trust London UK; ^5^ Department of Urology Chelsea and Westminster NHS Foundation Trust London UK; ^6^ Department of Urology University College London Hospitals NHS Foundation Trust London UK; ^7^ Department of Urology Guy's and St Thomas’ NHS Foundation Trust London UK; ^8^ Nightingale‐Saunders Clinical Trials and Epidemiology Unit, King's Clinical Trials Unit and Division of Methodology King's College London London UK; ^9^ Hamlyn Centre for Robotic Surgery, Department of Surgery and Cancer, Faculty of Medicine Imperial College London London UK; ^10^ Division of Surgical Interventions, Diagnostics and Device, Clinical Trials Research Unit University of Leeds Leeds UK

**Keywords:** prostate cancer, robotic surgery, radical prostatectomy, artificial intelligence, deep learning, fluorescence confocal microscopy, intraoperative margin analysis, positive surgical margins

## Abstract

**Objective:**

To develop and validate a deep learning model for interpretation of fluorescence confocal microscopy (FCM) images for intraoperative surgical margin assessment during radical prostatectomy (RP).

**Patients and Methods:**

Fluorescence confocal microscopy images from the multicentre Imperial Prostate 8‐Fluorescence Confocal Microscopy for Rapid Evaluation of Surgical Cancer Excision (IP8‐FLUORESCE) study were used to train and test a convolutional neural network model. The modified model incorporated focal loss with label smoothing, dropout regularisation, adaptive class weighting, and weighted sampling to address pronounced class imbalance. Images were pre‐processed by extracting regions of interest at a defined digital zoom level and normalised to 896 × 896 pixels. The reference standard was surgical margin status on conventional histopathology assessed by an expert histopathologist. Diagnostic performance was assessed using sensitivity, specificity, positive and negative predictive value, area under the receiver‐operating‐characteristic curve (AUC), and calibration via Brier scores. External validation was conducted using an independent dataset from the LaserSAFE feasibility trial. Model explainability was evaluated using Gradient‐weighted Class Activation Mapping (Grad‐CAM) and a custom graphical user interface (GUI) was developed to support real‐time deployment.

**Results:**

A total of 275 images (37 tumour and 238 benign from 24 patients) were included for model development and internal testing. On the internal test set (*n* = 57), the model achieved a sensitivity of 87.5%, specificity of 97.9%, and an AUC of 0.93, with good calibration (Brier score 0.16). External validation using 46 independent images yielded a sensitivity of 91.3%, specificity of 73.9%, and an AUC of 0.83, with acceptable calibration (Brier score 0.20). Grad‐CAM visualisations aligned with malignant structures on FCM images, and the GUI enabled rapid, interpretable predictions in <2 s.

**Conclusions:**

We developed and validated a deep learning model for interpretation of FCM images from RP specimens, which demonstrated strong discriminative performance and generalisability for automated FCM interpretation. This approach represents a scalable solution for real‐time intraoperative margin assessment and may reduce reliance on intraoperative pathology support.

**Abbreviations**
AI: artificial intelligence
AUC: area under the ROC curve
FCM: fluorescence confocal microscopy
Grad‐CAM: gradient‐weighted class activation mapping
GUI: graphical user interface
NeuroSAFE: Neurovascular Structure Adjacent Frozen‐Section Examination
PSM: positive surgical margin
(N)(P)PV: (negative) (positive) predictive value
ROC: receiver operating characteristic
RP: radical prostatectomy

## Introduction

Achieving negative surgical margins while preserving the neurovascular bundles remains one of the most important intraoperative challenges in radical prostatectomy (RP) for organ‐confined prostate cancer, as positive surgical margins (PSMs) may compromise oncological outcomes [1]. The Neurovascular Structure Adjacent Frozen‐Section Examination (NeuroSAFE) technique has demonstrated improved nerve‐sparing rates and postoperative erectile function without compromising oncological safety [2]. However, it is unlikely to be widely adopted due to substantial resource requirements, an unacceptable extension of operative time, and the need for an ‘on‐call’ histopathologist [3].

Fluorescence confocal microscopy (FCM) is a real‐time imaging method that enables intraoperative visualisation of prostate tissue at high resolution with minimal tissue processing and no specimen sectioning [4, 5, 6, 7]. Several studies have highlighted the feasibility of FCM in RP, and its diagnostic accuracy for clinically relevant PSMs is close to that of the NeuroSAFE technique [6, 8, 9, 10]. While FCM represents a more scalable alternative, it is still reliant on expert image interpretation by trained pathologists, in a time where the pathology workforce is in crisis [11].

Image analysis supported by artificial intelligence (AI) could address this limitation by enabling automated, real‐time classification of FCM images without the need for on‐site pathology expertise. In this study, we aimed to develop a deep learning model capable of classifying surgical margin status in FCM images from RP specimens.

## Patients and Methods

### Development and Interval Validation Dataset

We developed and externally validated a deep learning model using FCM images acquired during RP, aiming to assess its diagnostic accuracy for classifying images as containing PSMs or negative surgical margins. The model was developed using annotated FCM images from the Imperial Prostate 8‐Fluorescence Confocal Microscopy for Rapid Evaluation of Surgical Cancer Excision (IP8‐FLUORESCE) study, a prospective multicentre study evaluating the diagnostic accuracy of FCM for intraoperative surgical margin status in 156 RP specimens compared to final histology [10]. Consecutive men undergoing RP for treatment of localised or locally advanced prostate cancer were included; those undergoing salvage treatments were excluded. The FCM images were acquired using the Histolog® Scanner (SamanTree Medical SA, Lausanne, Switzerland) across three UK tertiary referral centres. Six anatomical margins of each prostate specimen were imaged *ex vivo* (apex, base, left and right posterolateral, anterior, and posterior) covering the entire surface of intact prostate specimens. The FCM images were interpreted and annotated by two expert uropathologists and compared against the matched surface on final histology. Limited clinical information (PSA, MRI, and prostate biopsy results) was provided to pathologists upon interpretation of FCM images and final histology.

Images were acquired from 29 patients. All FCM images containing PSMs that were confirmed as positive on histology (‘tumour’) were included. A random but representative sample of ‘benign’ images were included that captured the diverse tissue types (e.g., muscle, stroma, vessels, nerves, and fat) that can be found in negative cases. Only cases that were confirmed as true positives or negatives according to the ground truth (final histology) were included. Images were pre‐processed by capturing regions of interest at a digital zoom level of 70% of the Histolog Scanner's maximum display magnification, then normalised to 896 × 896 pixels for analysis. The resulting dataset was split into training (65%), internal validation (15%) and testing (20%) subsets at the image level.

### Model Development

The ResNet50 was selected as the base model. ResNet50 is an open‐source pre‐trained convolutional neural network‐based architecture with proven performance in medical image classification that can be robustly trained on small datasets [12, 13, 14]. While other models, such as transformer‐based architectures, were considered, these generally require substantially larger datasets for optimal performance and may risk overfitting when trained on limited medical imaging data. In addition, a significant class imbalance was present in our dataset due to the relatively limited event rate of PSMs in RP and that the entire prostate surface was scanned meaning there were more benign images than tumour. To improve performance on the highly imbalanced dataset, we modified the model to incorporate four key adaptations:

1. Improved focal loss with label smoothing to emphasise difficult tumour cases while reducing model overconfidence (γ = 3; smoothing factor 0.1).
2. Dropout regularisation within the classifier head (dropout rate 0.5) to prevent overfitting.
3. Enhanced class weighting using the ‘effective number of samples’ approach (β = 0.9999) to stabilise learning when one class dominates.
4. Weighted sampling during training to ensure adequate representation of tumour images in each training batch.

A probability threshold of 0.5 was used for binary classification between tumour and benign images. The modified model was termed Imbalanced‐ResNet50. Model development was performed in PyTorch 2.7 using pre‐trained ImageNet weights. The model was trained for up to 10 epochs (learning rate 2 × 10^−5^, batch size 8, weight decay 0.02) with early stopping after two epochs without improvement to prevent overfitting.

### External Validation

External validation was performed using images from the LaserSAFE feasibility trial [15]. Pseudonymised FCM images acquired from intact prostate specimens using the Histolog Scanner were used. All images were independently reviewed and annotated by two expert uropathologists. The dataset was entirely independent and from a separate patient population and acquisition team, ensuring unbiased assessment of model generalisability.

### Statistical Analysis

Sensitivity, specificity, positive predictive value (PPV), and negative predictive value (NPV) were calculated using confusion matrices and presented with 95% CIs using the Clopper‐Pearson exact method. Receiver‐operating‐characteristic (ROC) curves were used to assess discriminative performance. Calibration was assessed using reliability plots comparing predicted vs observed probabilities and quantified using the Brier score. Discrimination and calibration analyses were performed for internal testing and external validation datasets. Threshold analysis was performed at varying probability thresholds and reported with accuracy and F1 score. Analyses were performed in Python 3.11 (Python Software Foundation, Wilmington, DE, USA). The study was reported in accordance with the Standards for Reporting of Diagnostic Accuracy ‐ Artificial intelligence (STARD‐AI) recommendations.

### Model Explainability

To aid interpretability, the model's decision‐making process was visualised using gradient‐weighted class activation mapping (Grad‐CAM). Grad‐CAM generates a heatmap over the input image highlighting the regions that most strongly influenced the model's prediction [16]. For each correctly and incorrectly classified case, Grad‐CAM visualisations were generated from the final convolutional layer of the Imbalanced‐ResNet50 model and overlaid on the corresponding FCM images. These visualisations were reviewed qualitatively by an expert uropathologist to assess whether areas of high model attention corresponded to histologically malignant structures. This approach aimed to provide greater explainability for model outputs and help identify sources of incorrect classification.

### Graphical User Interface (GUI) Development

A custom GUI was developed in PyQt5 (Riverbank Computing Ltd, Poundbury, Dorchester, UK) to facilitate real‐time interaction with the trained model. The interface aimed to allow users to upload FCM images, perform classification using the Imbalanced‐ResNet50 model, and display a binary prediction (‘tumour’ or ‘benign’) with an associated probability score. The GUI was also designed to display the Grad‐CAM heatmap superimposed on the original image to highlight regions contributing to the prediction.

### Ethical Approval

Ethical approval was granted by the tissue biobanks at each study site.

## Results

In total, 275 images (37 tumour and 238 benign) from the IP8‐FLUORESCE dataset were used for model development and internal validation/testing, and class imbalance was maintained when splitting into subsets. Images were included from scans of any prostate surface (Table 1). Baseline characteristics are presented in Table 2.

**Table 1:** Images used per surface for model development, internal testing, and internal validation.

Surface	Number of images (%)
Posterolateral	66 (23.9)
Apex	58 (21.0)
Base	38 (13.8)
Anterior	50 (18.1)
Posterior	64 (23.2)
Total	276 (100.0)

**Table 2:** Baseline characteristics of the 29 included patients.

Characteristic	Value
Age, years, median (IQR)	64 (58–66)
PSA level, ng/mL, median (IQR)	7.6 (5.6–10.5)
Prostate volume, mL, median (IQR)	40.0 (30.8–46.5)
Charlson Comorbidity Index score, median (IQR)	4 (4–5)
Cambridge Prognostic Group, *n* (%)	
2	11 (37.9)
3	12 (41.4)
4	3 (10.3)
5	3 (10.3)
Final Gleason grade, *n* (%)	
3 + 4	18 (62.1)
4 + 3	8 (27.6)
4 + 5	3 (10.3)
Pathological T stage, *n* (%)	
T2a	2 (6.9)
T2c	5 (17.2)
T3a	16 (55.2)
T3b	6 (20.7)

### Internal Testing

On the internal test set (*n* = 57; eight tumour images, 49 benign images), the Imbalanced‐ResNet50 model achieved high diagnostic accuracy. Sensitivity for tumour detection was 87.5% (95% CI 47.4–99.7%) and specificity 97.9% (95% CI 89.2–99.9%). The corresponding PPV and NPV were 87.5% (95% CI 47.4–99.7%) and 97.9% (95% CI 89.2–99.9%), respectively, and the area under the ROC curve (AUC) was 0.93 (95% CI 0.80–1.00). Calibration on the internal test set was good (Brier score 0.16), showing minor overestimation at lower predicted probabilities but satisfactory alignment between predicted and observed outcomes at higher probabilities.

### External Validation

A total of 46 images from the LaserSAFE dataset were used for external validation (23 tumour, 23 benign). Only posterolateral surfaces were included as no other prostate margins were scanned in the LaserSAFE study. Here, the Imbalanced‐ResNet50 model achieved a similar sensitivity of 91.3% (95% CI 71.9–98.9%) but lower specificity (73.9%, 95% CI 51.6–89.8%). PPV and NPV were 77.8% (95% CI 57.7–91.4%) and 89.5% (95% CI 66.9–99.0%), respectively. Discriminative ability of the model on the external testing set was also slightly lower, resulting in an AUC of 0.83 (95% CI 0.72–0.93). The model demonstrated acceptable calibration (Brier score 0.20) on external validation, with mild overestimation at intermediate predicted probabilities but good alignment at higher values.

### Threshold Analysis

Diagnostic performance across different probability thresholds is shown in Tables 3 and 4. The pre‐defined threshold of 0.5 provided the best balance between sensitivity and specificity in both the internal test and external validation sets.

**Table 3:** Diagnostic performance of the model on the internal test set at varying probability thresholds.

Threshold	Sensitivity	Specificity	PPV	NPV	Accuracy	F1 score
0.30	1.000	0.469	0.235	1.000	0.544	0.381
0.40	1.000	0.898	0.615	1.000	0.912	0.762
0.50	0.875	0.980	0.875	0.980	0.965	0.875
0.60	0.875	0.980	0.875	0.980	0.965	0.875
0.70	0.625	1.000	1.00	0.942	0.947	0.769

**Table 4:** Diagnostic performance of the model on the external validation set at varying probability thresholds.

Threshold	Sensitivity	Specificity	PPV	NPV	Accuracy	F1 score
0.30	1.000	0.000	0.500	NA	0.500	0.667
0.40	1.000	0.130	0.535	1.000	0.565	0.697
0.50	0.913	0.739	0.778	0.895	0.826	0.840
0.60	0.391	1.000	1.000	0.622	0.696	0.563
0.70	0.000	1.000	NA	0.500	0.500	0.000

### Explainability and Workflow Integration

**Fig. 1:**
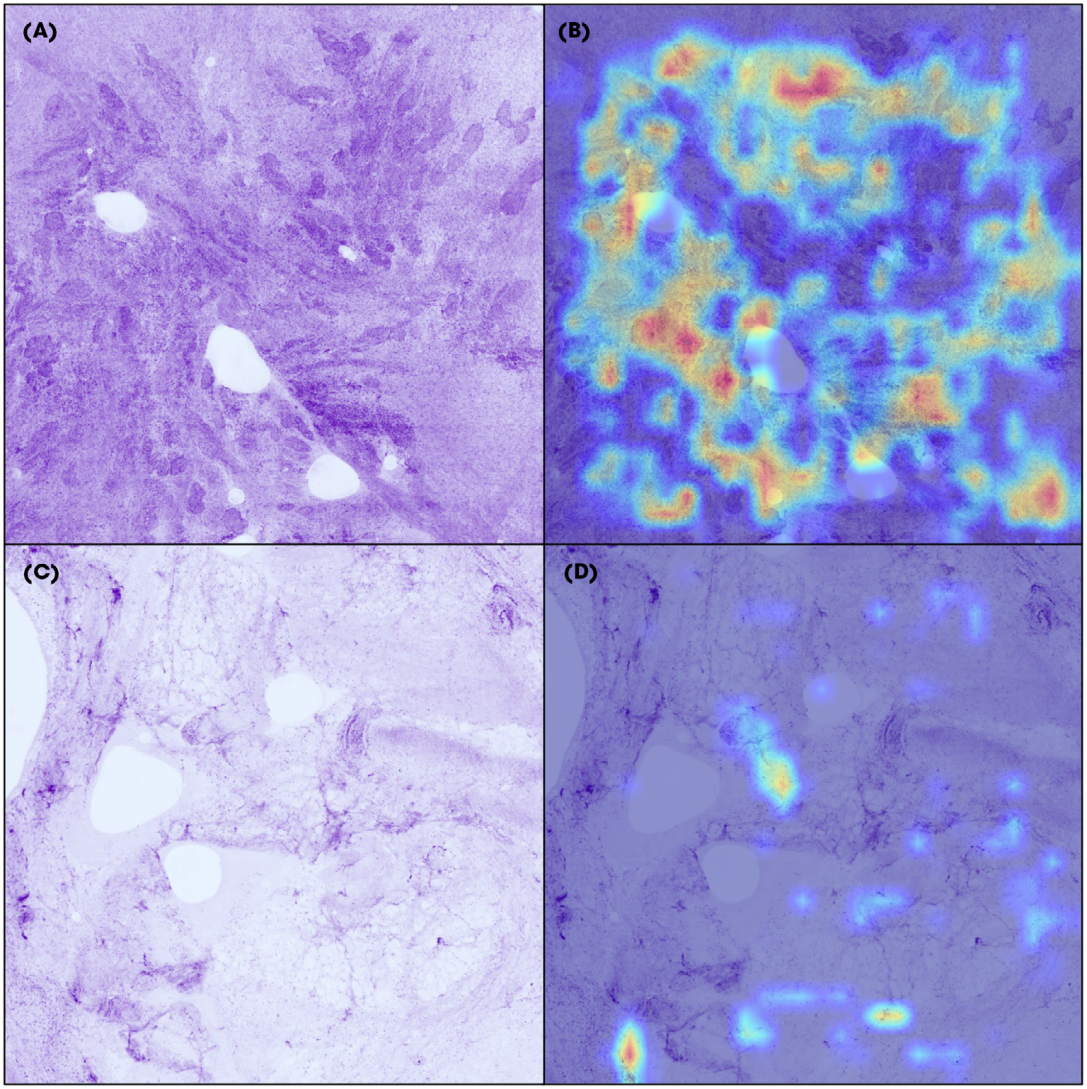
True positive (tumour) FCM image **(A)** with Grad‐CAM overlay **(B)** and true negative (benign) image **(C)** with Grade‐CAM overlay **(D)**.

The Grad‐CAM heatmaps consistently highlighted glandular and stromal regions corresponding to histologically confirmed prostate adenocarcinoma (Fig. 1).

**Fig. 2:**
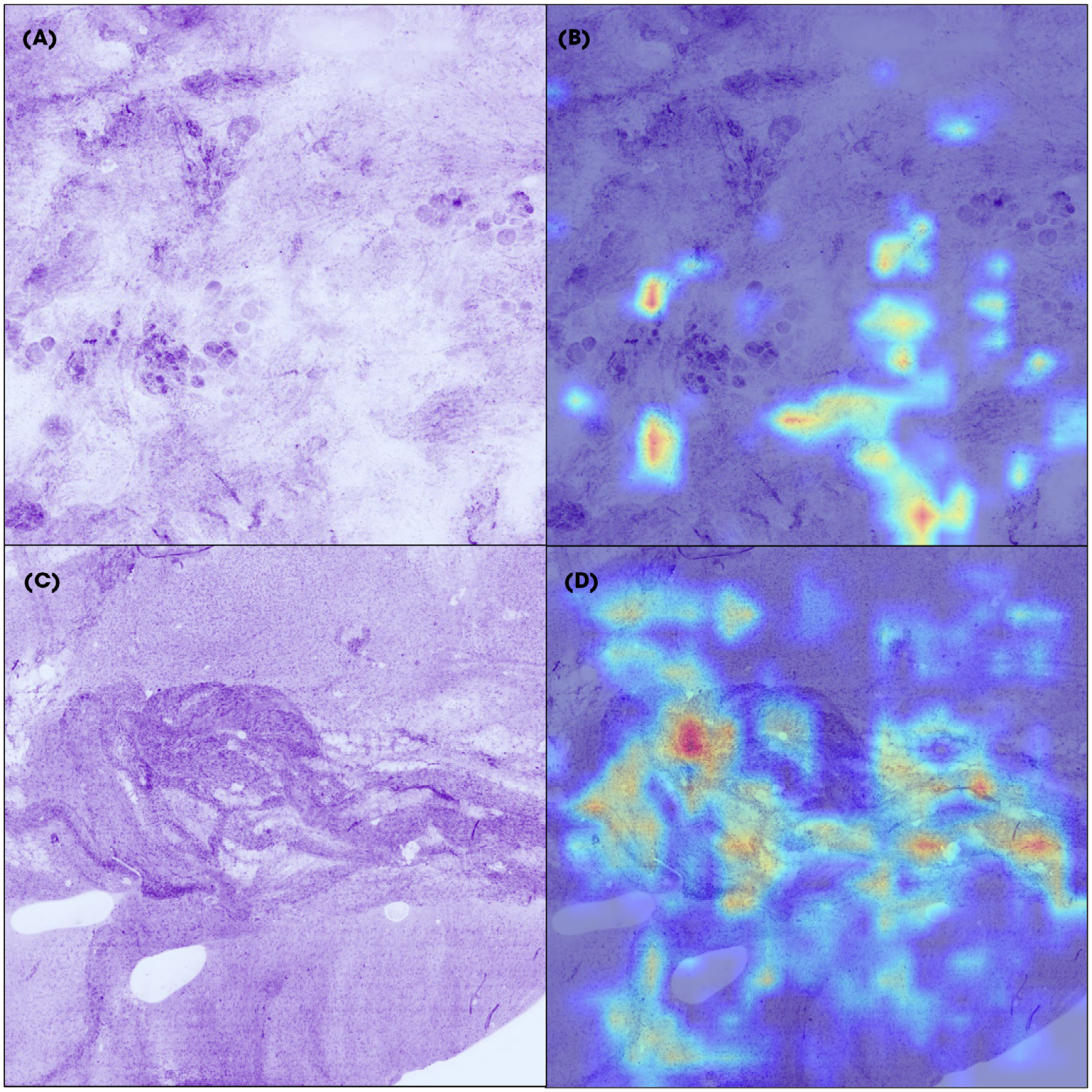
False positive case FCM image **(A)** with Grad‐CAM overlay **(B)** and false negative (benign) image **(C)** with Grade‐CAM overlay **(D)**.

Review of false‐positive cases using Grad‐CAM revealed model attention focused predominantly on closely packed neural structures, which on FCM can resemble crowded glandular architecture and thereby mimic malignant features. In the limited number of false‐negative cases, Grad‐CAM highlighted atypical glandular structures with a lobular arrangement that may be interpreted as benign, resulting in incorrect benign classification (Fig. 2).

**Fig. 3:**
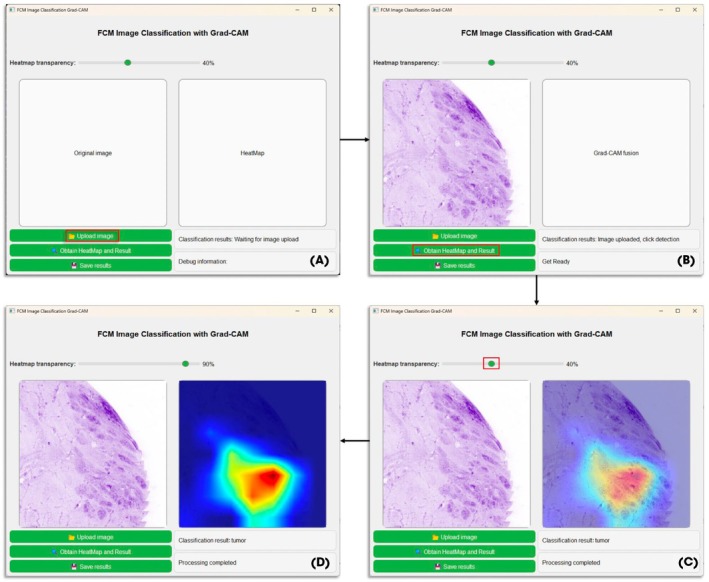
The GUI workflow with red boxes marking selected function: **(A)** shows initial interface with function buttons and information panel; **(B)** after uploading an image; **(C)** generated Grad‐CAM heatmap and classification result; and **(D)** heatmap transparency adjusted to 90%.

The GUI allowed rapid image upload, classification, and visualisation of Grad‐CAM overlays (Fig. 3). The mean inference time per image was <2 s on a standard workstation (NVIDIA RTX 3080 GPU, 12GB VRAM) and the binary outputs (‘tumour’ or ‘benign’) with associated probability scores were displayed in real time.

## Discussion

In this study, we report the development, internal testing, and validation of a deep learning model for interpretation of FCM images acquired from intact RP specimens. Using data from the multicentre IP8‐FLUORESCE study for model development with independent validation on the LaserSAFE trial dataset, our Imbalanced‐ResNet50 model achieved excellent diagnostic accuracy for detection of cancer (AUC 0.93), which remained high on external validation (AUC 0.83). These findings provide proof‐of‐concept that automated image analysis could facilitate intraoperative margin assessment without the need for on‐site pathology review.

Radical prostatectomy is the ‘gold standard’ surgical treatment option for men with localised or locally advanced prostate cancer, but PSMs occur in up to 40% of cases [17, 18, 19]. Not all PSMs are equal, but certain characteristics are associated with adverse medium‐ (biochemical recurrence, adjuvant treatment) and long‐term (metastasis‐free survival, prostate cancer‐specific and overall mortality) oncological outcomes [1]. Considerable efforts have been spent evaluating methods of intraoperative margin assessment that can offer men the best chance of a good functional outcome through neurovascular‐bundle preservation without compromising oncological safety from PSMs. Level 1 evidence for the efficacy of the NeuroSAFE approach was recently reported in the NeuroSAFE PROOF phase III randomised controlled trial (ClinicalTrials.gov identifier: NCT03317990), which compared NeuroSAFE‐guided robot‐assisted RP with standard nerve‐sparing surgery across five UK centres [2]. At 12 months, the mean five‐item version of the International Index of Erectile Function (IIEF‐5) score was significantly higher in the NeuroSAFE group, indicating superior postoperative erectile function recovery. Early urinary continence also improved at 3 months, without a significant increase in PSM rate. The erectile function benefit was enhanced in patients who would not otherwise have undergone bilateral nerve‐sparing in usual practice. Yet despite this high‐level evidence, NeuroSAFE is unlikely to be widely adopted. The initial outlay for equipment such as cryotomes is high and the technique requires complex tissue processing, which has limited its use to a handful of high‐volume centres. The additional operating time may also be considered unacceptable, with NeuroSAFE typically adding 45–60 min to a robot‐assisted RP [20]. Several novel techniques for intraoperative margin assessment have been described, such as optical coherence tomography, photodynamic diagnosis, light reflectance spectroscopy, and specimen microscopic positron emission tomography CT, with reported sensitivities and specificities varying widely [21]. FCM is at the forefront of these technologies and addresses several of the limitations inherent to froze section. Using a mobile confocal microscope in the operating room requires little to no tissue preparation and no skilled laboratory staff and was shown in the IP8‐FLUORESCE study to have comparable diagnostic accuracy to NeuroSAFE for ‘clinically significant’ PSMs (i.e., those ≥3 mm in length) [10]. However, until now, interpretation of FCM images has remained within the remit of the pathologist. AI tools such as that described here represent a potential solution to the reliance on highly trained pathologists.

To the best of our knowledge, our study is the first to report the use of an AI‐based model for interpretation of FCM images in RP for surgical margin assessment. Our Imbalanced‐ResNet50 model was designed specifically for the class imbalance inherent in prostate margin datasets, in which benign tissue predominates. Through the introduction of improved focal loss with label smoothing, adaptive class weighting, dropout regularisation, and weighted sampling, the model maintained strong sensitivity for tumour detection with a high specificity. These modifications likely contributed to the model's preserved sensitivity on the independent external validation dataset. However, specificity was lower on external validation with a rise in false positives, possibly explained by the fact that the model was trained on images from all prostate surfaces, whereas the external validation cohort consisted entirely of images from the posterolateral surfaces. False positives must be minimised as far as possible to reduce the risk of unnecessary secondary resection of the neurovascular bundles leading to poorer erectile function. Analysis of false‐positive predictions on the external validation dataset showed that all misclassified benign images were associated with probability scores close to the decision threshold (range 0.39–0.55), indicating low model confidence rather than gross misclassification. These borderline cases likely reflect structural overlap between benign fibromuscular stroma and tumour regions in FCM, where image contrast and glandular crowding can mimic malignancy. From a clinical perspective, this behaviour is reassuring, as it suggests that the model acts conservatively at intermediate probabilities rather than making high‐confidence false predictions. We did not undertake formal threshold optimisation due to the limited number of true positive tumour cases within the development and validation datasets. Future work using larger cohorts is needed to determine appropriate probability cut‐offs for clinical use. We anticipate the use of automated reading tools as real‐time decision aids for the trained urologist. A direct review of the images by a urologist should always be undertaken. In cases where a decision about the presence of significant margin cannot be reached, a remote opinion from a pathologist can be sought.

We enhanced model explainability using Grad‐CAM visualisations, which generate heatmaps showing the regions of each FCM image that most influenced the model's prediction. In our analysis, Grad‐CAMs consistently highlighted glandular and stromal areas corresponding to histologically confirmed tumour, indicating that the model is learning clinically relevant features rather than relying on artefactual patterns. The prototype GUI developed for this study further demonstrates the feasibility of integrating the model into a real‐time intraoperative workflow, with both image classification and Grad‐CAM generation performed within seconds. This feature facilitates immediate user‐friendly feedback on margin status following image acquisition.

This study has several limitations. First, both the testing and external validation datasets were small, with limited numbers of positive cases, limiting the precision of performance estimates. Larger, prospective multicentre datasets will be required to confirm generalisability. Second, image‐level classification was used rather than pixel‐level segmentation, which may limit the ability to localise microscopic foci of margin involvement. Linking FCM images directly with spatially registered histopathology could enable more granular annotation and training. Third, although the model demonstrated good interpretability through Grad‐CAM patterns, these assessments were qualitative. Future studies could incorporate quantitative explainability metrics and human–AI reader studies to evaluate concordance between model attention and expert interpretation. Finally, while there is no single accepted definition of a ‘clinically significant’ surgical margin, PSM length >3 mm is generally considered to be a poorer prognostic marker. The correlation between surface area of a PSM when RP specimens are scanned *en face* with oncological outcomes should be explored.

Despite these limitations, the present work provides an important step toward scalable intraoperative image analysis in RP. Future directions include multicentre validation across diverse populations and integration with digital operating systems that could eventually enable near‐real‐time, AI‐assisted surgical decision support. Beyond prostate cancer, similar frameworks could be adapted for other organ systems where intraoperative tissue assessment could inform surgical decision‐making, such as partial nephrectomy or breast‐conserving surgery.

## Conclusion

We report the development, internal testing, and external validation of Imbalanced‐ResNet50, a deep learning model designed to classify surgical margin status on FCM images from RP specimens. The model demonstrated strong discriminative performance with acceptable calibration, and performance remained robust on an independent external dataset. These findings suggest that automated interpretation of FCM is both feasible and scalable, with the potential to improve surgical outcomes while reducing reliance on scarce pathology resources.

## Disclosure of Interests

Nothing is declared.
